# Fibrosis signature of anastomotic margins for predicting anastomotic stenosis in rectal cancer with neoadjuvant chemoradiotherapy and sphincter-preserving surgery

**DOI:** 10.1093/gastro/goae012

**Published:** 2024-03-19

**Authors:** Zhun Liu, Meifang Xu, Qian Yu, Jianyuan Song, Qili Lin, Shenghui Huang, Zhifen Chen, Ying Huang, Pan Chi

**Affiliations:** Department of Colorectal Surgery, Fujian Medical University Union Hospital, Fuzhou, Fujian, P. R. China; Department of Pathology, Fujian Medical University Union Hospital, Fuzhou, Fujian, P. R. China; Department of Pathology, Fujian Medical University Union Hospital, Fuzhou, Fujian, P. R. China; Department of Radiation Oncology, Fujian Medical University Union Hospital, Fuzhou, Fujian, P. R. China; Department of Pathology, Fujian Medical University Union Hospital, Fuzhou, Fujian, P. R. China; Department of Colorectal Surgery, Fujian Medical University Union Hospital, Fuzhou, Fujian, P. R. China; Department of General Surgery, Fujian Medical University Union Hospital, Fuzhou, Fujian, P. R. China; Department of Colorectal Surgery, Fujian Medical University Union Hospital, Fuzhou, Fujian, P. R. China; Department of General Surgery, Fujian Medical University Union Hospital, Fuzhou, Fujian, P. R. China; Department of Colorectal Surgery, Fujian Medical University Union Hospital, Fuzhou, Fujian, P. R. China; Department of General Surgery, Fujian Medical University Union Hospital, Fuzhou, Fujian, P. R. China; Department of Colorectal Surgery, Fujian Medical University Union Hospital, Fuzhou, Fujian, P. R. China; Department of General Surgery, Fujian Medical University Union Hospital, Fuzhou, Fujian, P. R. China

**Keywords:** rectal cancer, radiation-induced colorectal fibrosis, anastomotic stenosis, nomogram

## Abstract

**Background:**

Radiation-induced colorectal fibrosis (RICF) is a common pathological alteration among patients with rectal cancer undergoing neoadjuvant chemoradiotherapy (nCRT). Anastomotic stenosis (AS) causes symptoms and negatively impacts patients’ quality of life and long-term survival. In this study, we aimed to evaluate the fibrosis signature of RICF and develop a nomogram to predict the risk of AS in patients with rectal cancer undergoing nCRT.

**Methods:**

Overall, 335 pairs of proximal and distal margins were collected and randomly assigned at a 7:3 ratio to the training and testing cohorts. The RICF score was established to evaluate the fibrosis signature in the anastomotic margins. A nomogram based on the RICF score for AS was developed and evaluated by using the area under the curve, decision curve analysis, and the DeLong test.

**Results:**

The training cohort included 235 patients (161 males [68.51%]; mean age, 59.61 years) with an occurrence rate of AS of 17.4%, whereas the testing cohort included 100 patients (72 males [72.00%]; mean age, 57.17 years) with an occurrence rate of AS of 11%. The RICF total score of proximal and distal margins was significantly associated with AS (odds ratio, 3.064; 95% confidence interval [CI], 2.200–4.268; *P *<* *0.001). Multivariable analysis revealed that the RICF total score, neoadjuvant radiotherapy, and surgical approach were independent predictors for AS. The nomogram demonstrated good discrimination in the training cohort (area under the receiver-operating characteristic curve, 0.876; 95% CI, 0.816–0.937), with a sensitivity of 68.3% (95% CI, 51.9%–81.9%) and a specificity of 85.5% (95% CI, 78.7%–89.3%). Similar results were observed in the testing cohort.

**Conclusions:**

This study results suggest that the RICF total score of anastomotic margins is an independent predictor for AS. The prediction model developed based on the RICF total score may be useful for individualized AS risk prediction in patients with rectal cancer undergoing nCRT and sphincter-preserving surgery.

## Introduction

Radiation-induced colorectal fibrosis (RICF) is a common pathological alteration among patients with rectal cancer undergoing neoadjuvant chemoradiotherapy (nCRT) [1]. Excessive collagen in the intestinal wall can lead to decreased intestinal compliance and an increased risk of low anterior resection syndrome and anastomotic stenosis (AS) [2, 3]. AS causes symptoms such as abdominal distension and pain, and negatively impacts patients’ quality of life and long-term survival [4, 5]. Previous models have primarily focused on clinical characteristics; however, the relationship between AS and fibrosis in anastomotic margins has not been thoroughly investigated.

The incidence of AS after rectal cancer surgery ranges from 2.0% to 22% [6]. Currently, membranous stenosis is typically treated with anal dilatation [7]. However, tubular stenosis with fibrosis may require more invasive interventions, such as endoscopic balloon dilation or tract reconstruction, which increase the risk of perforation, pelvic infection, bleeding, and even permanent stoma [8, 9]. Preoperative radiotherapy increases the risk of anastomotic leakage and stenosis after rectal cancer sphincter-preserving surgery [10]. Furthermore, radiotherapy causes damage to the colorectal tissue, leading to edema, inflammation of the mucosal and submucosal layers, and ulcers, ultimately resulting in intestinal fibrosis, which is one of the primary causes of AS [11, 12]. Our previous study showed that patients with AS who received nCRT had a distinct pathological hallmark of excessive collagen in the anastomotic site [13]. However, the fibrosis feature of anastomosis margins in patients with rectal cancer receiving nCRT remains poorly understood [14–16]. Therefore, identifying risk factors for AS in rectal cancer is crucial for advancing personalized prediction and management. Moreover, incorporating fibrosis features of anastomotic margins, along with clinical characteristics, holds promise for improving the prediction of AS in patients with rectal cancer undergoing nCRT and sphincter-preserving surgery.

Thus, in this study, we aimed to assess the fibrosis features of RICF in proximal and distal margins, and develop a nomogram based on the RICF score to distinguish genuine high-risk AS in patients with rectal cancer undergoing nCRT and sphincter-preserving surgery.

## Patients and methods

### Patients

Overall, 335 pairs of proximal and distal margins were collected from patients with rectal cancer who underwent nCRT and sphincter-preserving surgery (for surgical procedures, see Supplementary Appendix S1) at Fujian Medical University Union Hospital (FMUUH) between July 2012 and January 2021. The samples were randomly categorized into training and testing cohorts at a 7:3 ratio (Supplementary Figure S1). The inclusion criteria were as follows: (i) histologically confirmed rectal adenocarcinoma; (ii) sphincter-preserving surgery; (iii) straight end-to-end colorectal or coloanal anastomosis using the double-stapling technique; (iv) treatment-naive patient with rectal cancer undergoing nCRT; and (v) negative anastomotic doughnuts. The exclusion criteria were as follows: (i) inflammatory bowel diseases, such as Crohn’s disease or ulcerative colitis; (ii) history of abdominal surgery; (iii) palliative resection; (iv) post-operative pelvic radiotherapy; (v) post-operative anastomotic recurrence; and (vi) post-operative survival of <24 months. The ethics committee of FMUUH approved this study.

### Characteristics and outcomes

Baseline clinical characteristics were collected, including age, body mass index, sex, smoking and drinking habits, diabetes, hypertension, ypTNM stage, tumor regression grade, interval time from radiotherapy to surgery, tumor location, surgical approach, ileostomy, length of surgery, and intraoperative bleeding. The 8th edition of the American Joint Committee on Cancer Staging Manual was used to determine the post-operative pathological tumor Tumor Node Metastasis (TNM) classification.

AS was defined as significant tubular stenosis of the anastomotic site observed post-operatively, including stenosis of the proximal bowel segment of the anastomosis and other regions, which met any of the following criteria: (i) inability to pass an index finger; (ii) inability to pass a 12-mm colonoscope; and (iii) magnetic resonance imaging or proctography reveals AS [17, 18]. Anastomotic leakage was defined as intestinal wall communication due to compromised surgical connection integrity. It encompasses evident clinical signs and subtler subclinical forms identified through imaging, clinical assessment, or exploratory surgery [19]. Post-operative anastomotic sites of all patients with rectal cancer were followed up for 2 years.

### Evaluation of radiation-induced colorectal fibrosis

**Figure 1.**
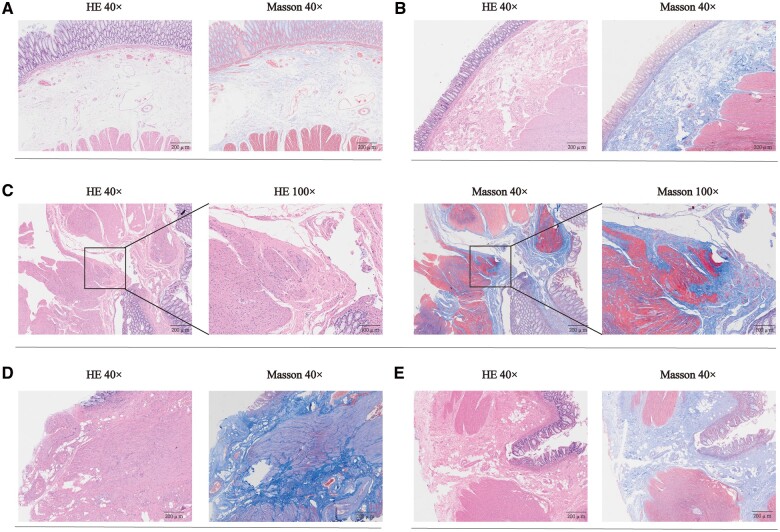
Fibrosis signature of radiation-induced colorectal fibrosis (RICF). The RICF scores from 0 to 4 were analysed using Masson trichrome staining compared with those obtained using hematoxylin-eosin staining. (A) For RICF score = 0, no obvious or mild fibrosis; (B) for RICF score = 1, obvious fibrosis in the submucosa or subserosa; (C) for RICF score = 2, fibrosis replaces part of the muscular propria; (D) for RICF score = 3, septa through muscularis propria, increase in subserosal collagen; (E) for RICF score = 4, significant transmural scar, marked subserosal collagen. 40× scale: 500 μm; 100× scale: 100 μm.

Histological analysis was performed on the proximal and distal margins of 335 patients with rectal cancer who underwent nCRT and sphincter-preserving surgery. The anastomotic distal and proximal “doughnut” specimens were harvested, fixed in 4% paraformaldehyde, dehydrated, and embedded in paraffin. Subsequently, the samples were sectioned (3 µm) using a microtome and stained with hematoxylin-eosin and Masson’s trichrome following standard protocols. The fibrosis signatures were observed in both the proximal and distal margins, and the RICF score was established as follows: RICF = 0 for no fibrosis, RICF = 1 for obvious fibrosis in the submucosa or subserosa, RICF = 2 for fibrosis replacing part of the muscular propria, RICF = 3 for septa through the muscular propria and an increase in subserosa collagen, and RICF = 4 for significant transmural scar and marked subserosa collagen (Figure 1). Two experienced independent gastrointestinal pathologists (M.X. and Q.Y.) who were blinded to all clinical data assessed the fibrosis signatures in the proximal and distal margins using the RICF score. Specific scoring systems for the morphological features of RICF in anastomotic margins are elaborated on in Supplementary Table S1. Notably, the RICF total score was determined by adding the RICF scores of both the proximal and distal margins.

### Prediction model development and evaluation

In the training cohort, both the 22 clinical characteristics and the fibrosis signature were subjected to univariate analysis to explore their association with AS, and variables with *P *<* *0.05 were selected for the multivariable analysis. Variables with *P *<* *0.05 in multivariable analysis were identified as independent predictor factors. The multivariable model was assessed for multicollinearity using tolerance and variance inflation factors, and effect modification was also evaluated. An independent predictor-based nomogram was constructed and the area under the receiver-operating characteristic curve (AUROC) was measured to quantify its discrimination. Moreover, the calibration of the nomogram was evaluated using a calibration curve and the Hosmer–Lemeshow test to assess its goodness of fit. In this prediction model development and validation study, we followed the Transparent Reporting of a multivariable prediction model for Individual Prognosis Or Diagnosis (TRIPOD) statement [20].

To assess the clinical utility of the nomogram, we used decision curve analysis (DCA) to evaluate the net benefits of the prediction model across various threshold probabilities. The optimal cut-off value was determined by selecting the maximum Youden index and assessing the sensitivity and specificity of the prediction model.

### Statistical analysis

Statistical analysis was performed using R software, version 3.6.3 (R Foundation for Statistical Computing) and IBM SPSS Statistics for Windows, version 23 (IBM Corp., Armonk, NY, USA). An independent-sample, unpaired, two-tailed *t*-test, or Mann–Whitney *U* test, as appropriate, was employed to evaluate the differences in continuous variables. Meanwhile, a χ^2^ test or Fisher exact probability test was used to compare the differences between categorical variables. A multivariable logistic regression analysis was conducted to estimate the odds ratio (OR) with a 95% confidence interval (CI) and identify the independent AS predictors. Differences with a two-sided *P *<* *0.05 were regarded as statistically significant.

## Results

### Participants

The training cohort comprised 235 patients (161 males [68.51%]; mean [standard deviation (SD)] age, 59.61 [11.16] years), with an AS rate of 17.4% (*n *=* *41). The testing cohort included 100 patients (72 males [72.00%]; mean [SD] age, 57.17 [12.46] years), with an AS rate of 11% (*n *=* *11). The median interval between AS diagnosis and rectal anterior resection was 8 (range, 4–24) months. The initial treatment for 52 patients with AS was as follows: 32 patients (61.5%) underwent endoscopic balloon dilatation, 1 (1.9%) received stent placement, 8 (15.5%) underwent transanal minimally invasive surgery, 10 (19.2%) underwent permanent stoma, and 1 (1.9%) had tract reconstruction. The patient characteristics of both cohorts are presented in Table 1. No statistically significant difference was found in AS prevalence between the training and testing cohorts (OR, 1.791; 95% CI, 0.840–3.482; *P *=* *0.168). Notably, the clinical characteristics were similar between the training and testing cohorts (all *P *>* *0.05) (Supplementary Table S2).

**Table 1.** Comparison of clinical characteristics of patients with AS in training and testing cohort

	Training cohort (*n *=* *235)			Testing cohort (*n *=* *100)		
Characteristic	With AS, *n *=* *41 (%)	Without AS, *n *=* *194 (%)	*P*	With AS, *n *=* *11 (%)	Without AS, *n *=* *89 (%)	*P*
Age (<65/≥65 years)	31 (75.61)/10 (24.39)	119 (61.34)/75 (38.66)	0.107	10 (90.9)/1 (9.1)	33 (37.1)/56 (62.9)	0.092
Sex (male/female)	35 (85.37)/6 (14.63)	126 (64.95)/68 (35.05)	0.010	10 (90.9)/1 (9.1)	62 (69.7)/27 (30.3)	0.175
BMI (≥25/<25)	9 (21.95)/32 (78.05)	69 (35.57)/125 (64.43)	0.103	1 (9.1)/10 (90.9)	31 (34.8)/58 (65.2)	0.087
Smoking	21 (51.22)/20 (48.78)	67 (34.54)132 (68.04)	0.052	7 (63.6)/4 (36.4)	41 (46.1)/48 (53.9)	0.345
Drinking	16 (39.02)/25 (60.98)	70 (36.08)/124 (63.92)	0.724	8 (72.7)/3 (27.3)	38 (42.7)/51 (57.3)	0.106
Diabetes	2 (4.88)/39 (95.12)	16 (8.25)/178 (91.75)	0.746	0 (0.0)/11 (100.0)	11 (12.4)/78 (87.6)	0.605
Hypertension	7 (17.07)/34 (82.93)	39 (20.10)/155 (79.90)	0.829	2 (18.2)/9 (81.8)	21 (23.6)/68 (76.4)	0.687
pT stage (T0–2/T3–4)	18 (43.9)/23 (56.1)	91 (46.9)/103 (51.3)	0.863	7 (63.6)/4 (36.4)	47 (52.8)/42 (47.2)	0.541
pN stage (N0/N+)	31 (75.6)/10 (24.4)	145 (74.7)/49 (25.3)	0.907	9 (81.8)/2 (18.2)	74 (83.1)/15 (16.9)	0.912
M stage (M0/M1)	37 (90.2)/4 (9.8)	182 (93.8)/12 (6.2)	0.491	11 (100.0)/0 (0.0)	78 (87.6)/11 (12.4)	0.605
AJCC stage (0–II/III–IV)	30 (73.2)/11(26.8)	142 (73.2)/52 (26.8)	0.997	9 (81.8)/2 (18.2)	64 (71.9)/25 (28.1)	0.722
TRG (0/1–3)	5 (12.2)/36 (87.8)	41 (21.1)/153 (78.95)	0.278	5 (45.5)/6 (54.5)	19 (21.3)/70 (78.7)	0.127
Neoadjuvant chemotherapy (XELOX/others)	27 (65.85)/14 (34.15)	146 (75.26)/48 (24.74)	0.243	6 (54.5)/5 (45.5)	64 (71.9)/25 (28.1)	0.298
Neoadjuvant radiotherapy (50.4 Gy, 25 F/25 Gy, 5 F)	40 (97.56)/1 (2.44)	149 (76.80)/45 (23.20)	0.001	11 (100.0)/0 (0.0)	71 (79.8)/18 (20.2)	0.206
Interval time from radiotherapy to surgery (<10/≥10 weeks)	14 (34.15)/27 (65.85)	26 (13.40)/168 (86.60)	0.003	3 (27.3)/8 (72.7)	6 (6.7)/83 (93.3)	0.058
Tumor location (low/mid–upper)	30 (73.17)/11 (26.83)	110 (56.70)/84 (43.30)	0.056	9 (81.8)/2 (18.2)	54 (60.7)/35 (39.3)	0.205
Intraoperative bleeding, mL (mean ± SD)	53 ± 17	70 ± 133	0.431	71 ± 23	56 ± 34	0.148
Length of surgery, min (mean ± SD)	248.4 ± 25.09	266.7 ± 77.10	0.131	251.8 ± 23.91	260.5 ± 69.44	0.681
Total lymph nodes harvested (<12/≥12)	15 (36.59)/26 (63.41)	74 (38.14)/120 (61.86)	0.852	5 (45.5)/6 (54.5)	50 (56.2)/39 (43.8)	0.536
Surgical approach (open/MIS)	13 (31.71)/28 (68.29)	7 (3.61)/187 (96.39)	0.001	0 (0.0)/11 (100.0)	4 (4.5)/85 (95.5)	0.473
Ileostomy	19 (46.34)/23 (53.66)	106 (54.64)/88 (45.36)	0.390	6 (54.5)/5 (45.5)	55 (61.8)/34 (38.2)	0.747
Anastomotic leakage	11 (26.83)/30 (73.17)	27 (13.91)/167 (86.09)	0.059	5 (45.5)/6 (54.5)	6 (6.7)/83 (93.3)	0.002

BMI = body mass index, SD = standard deviation, TRG = tumor regression grade, AJCC = American Joint Committee on Cancer, AS = anastomotic stenosis, MIS = minimally invasive surgery.

### RICF severity assessment in proximal and distal margins

According to the RICF score, the distal margins exhibited a higher grade than the proximal margins in the training cohort (2.2 ± 0.6 vs 1.7 ± 0.8, *P *<* *0.001); this finding was consistently observed in the testing cohort (*P *=* *0.045) and among all patients (*P *<* *0.001). As illustrated in Table 2, a significant difference was observed in the RICF score between the proximal and distal margins in patients with and without AS in both the training and testing cohorts (*P *<* *0.05). These findings were further confirmed in the testing cohort (*P *<* *0.05) and among all patients (all *P *<* *0.05). The RICF score of the proximal and distal margins was used to predict the risk of AS in patients with rectal cancer who underwent nCRT and sphincter-preserving surgery.

**Table 2.** RICF score of proximal and distal margins in patients with AS

	Training cohort			Testing cohort		
Variable	AS, *n *=* *41	No AS, *n *=* *194	*P*	AS, *n *=* *11	No AS, *n *=* *89	*P*
Proximal margins	2.4 ± 0.7	1.5 ± 0.8	0.001	2.2 ± 0.8	1.4 ± 0.8	0.004
Distal margins	2.7 ± 0.7	2.1 ± 0.6	0.001	2.5 ± 0.9	2.0 ± 0.5	0.016
RICF total score	5.1 ± 1.2	3.6 ± 1.1	0.001	4.6 ± 1.6	3.4 ± 1.0	0.001

RICF total score: the sum of the proximal and distal margins RICF scores.

RICF = radiation-induced colorectal fibrosis, AS = anastomotic stenosis.

The RICF total score of both proximal and distal margins showed a high predictive value for AS, with AUROC values of 0.817 (95% CI, 0.738–0.896), 0.802 (95% CI, 0.679–0.925), and 0.815 (95% CI, 0.748–0.882) in the training cohort, testing cohort, and all 335 patients, respectively. In the training cohort, a cut-off value of 4 was chosen as the maximum Youden index, yielding a sensitivity of 68.3% (95% CI, 51.9%–81.9%) and a specificity of 85.5% (95% CI, 78.7%–89.3%). Similarly, in the testing cohort, the sensitivity and specificity were 54.6% (95% CI, 23.4%–83.3%) and 85.4% (95% CI, 76.3%–92.0%), respectively. Overall, among all 335 patients, the sensitivity and specificity were 65.4% (95% CI, 50.9%–78.0%) and 84.8% (95% CI, 80.1%–88.8%), respectively. In conclusion, the RICF total score of proximal and distal margins is a useful predictor of AS in patients with rectal cancer who undergo nCRT and sphincter-preserving surgery.

### Prediction model development

**Figure 2.**
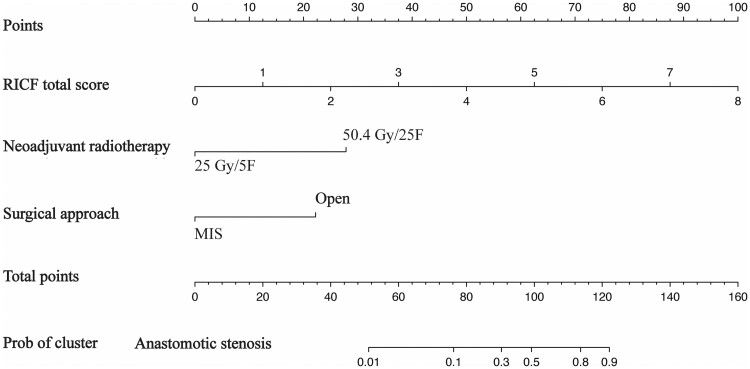
Nomogram indicating the risk of AS in patients with rectal cancer who are undergoing neoadjuvant chemoradiotherapy and sphincter-preserving surgery. For clinical use, the RICF total score is determined by drawing a line straight up to the point axis to establish the score associated with the differentiation. Next, this process is repeated for the other two covariates (neoadjuvant radiotherapy and surgical approach). The scores of each covariate are added and the total score is located on the total-score-point axis. Finally, a line is drawn straight down to the risk-of-AS axis to obtain the probability. F = fractions.

In this study, seven variables with a *P *<* *0.05 in univariate analysis were included in the multivariate logistic regression analysis to identify independent risk factors. As presented in Table 3, the results showed that neoadjuvant radiotherapy (OR, 13.496; 95% CI, 1.172–155.394; *P *=* *0.037), surgical approach (OR, 8.874; 95% CI, 2.482–31.736; *P *=* *0.001), and the RICF total score (OR, 3.189; 95% CI, 2.066–4.922; *P *<* *0.001) were independent predictors for AS. The variance inflation factor of each predictor was <10, while the corresponding tolerance was <0.1, indicating no multicollinearity among these predictors (Supplementary Table S3). A nomogram was developed by integrating these three independent predictors, as shown in Figure 2.

**Table 3.** Univariate and multivariable logistic regression for AS in the training cohort

	Univariate analysis		Multivariable analysis	
Variable	OR (95% CI)	*P*	OR (95% CI)	*P*
Age (<65/≥65 years)	1.954 (0.905–4.216)	0.088		
Sex (male/female)	3.148 (1.261–7.8.59)	0.014	NA	NA
BMI (<25/≥25)	1.963 (0.886–4.350)	0.097		
Smoking	1.990 (1.008–39.29)	0.047	NA	NA
Drinking	1.134 (0.567–2.266)	0.722		
Diabetes	0.571 (0.126–2.583)	0.466		
Hypertension	0.818 (0.337–1.985)	0.657		
pT stage (T0–2/T3–4)	0.886 (0.450–1.745)	0.726		
pN stage (N0/N+)	1.048 (0.479–2.292)	0.907		
M stage (M0/M1)	0.610 (0.186–1.996)	0.414		
AJCC stage (0–II/III–IV)	1.001 (0.468–2.142)	0.997		
TRG (0/1–3)	0.518 (0.191–1.404)	0.196		
Neoadjuvant chemotherapy (XELOX/others)	0.634 (0.308–1.307)	0.217		
Neoadjuvant radiotherapy (50.4 Gy, 25 F/25 Gy, 5 F)	12.08 (1.615–90.353)	0.015	13.496 (1.172–155.394)	0.037
Interval time from radiotherapy to surgery (<10/≥10 weeks)	3.350 (1.557–7.210)	0.002	NA	NA
Tumor location (low/mid–upper)	2.083 (0.978–4.396)	0.054		
Intraoperative bleeding, mL (mean ± SD)	0.998 (0.992–1.004)	0.473		
Length of surgery, min (mean ± SD)	0.995 (0.989–1.001)	0.119		
Total lymph nodes harvested (<12/≥12)	0.936 (0.465–1.881)	0.852		
Surgical approach (open/MIS)	12.40 (4.558–33.75)	0.001	8.874 (2.482–31.736)	0.001
Ileostomy	0.717 (0.365–1.409)	0.335		
Anastomotic leakage	2.685 (1.071–5.055)	0.045	NA	NA
RICF total score	3.642 (2.349–5.102)	0.001	3.189 (2.066–4.922)	0.001

BMI = body mass index, SD = standard deviation, TRG = tumor regression grade, AJCC = American Joint Committee on Cancer, AS = anastomotic stenosis, MIS = minimally invasive surgery, RICF = radiation-induced colorectal fibrosis, OR = odds ratio, CI = confidence interval.

### Clinical application

The nomogram based on the RICF score to predict the risk of AS exhibited excellent performance, with AUROC values of 0.876 (95% CI, 0.816–0.937) and 0.818 (95% CI, 0.790–0.928) in the training and testing cohorts, respectively (Supplementary Figure S2A and B). The calibration curve demonstrated an excellent agreement between the estimated probability of AS from the nomogram and the actual AS rate in both the training and testing cohorts (Supplementary Figure S2C and D). The Hosmer–Lemeshow test yielded a *P-*value of 0.792, indicating no deviation from a good fit. In the training cohort, a cut-off value of 0.612 was selected as the maximum Youden index, yielding sensitivity, specificity, accuracy, positive predictive value, and negative predictive value of 75.6% (95% CI, 59.7%–87.6%), 85.6% (95% CI, 79.8%–90.2%), 83.8%, 52.5%, and 94.3%, respectively. The testing cohort had sensitivity, specificity, accuracy, positive predictive value, and negative predictive value of 54.6% (95% CI, 23.4%–83.3%), 85.4% (95% CI, 76.3%–92.0%), 82.0%, 31.6%, and 93.8%, respectively. Among the 335 patients, the sensitivity, specificity, accuracy, positive predictive value, and negative predictive value were 71.2%, 85.5%, 83.8%, 47.4%, and 94.2%, respectively.

### Comparison with the clinical model for predicting AS

**Figure 3.**
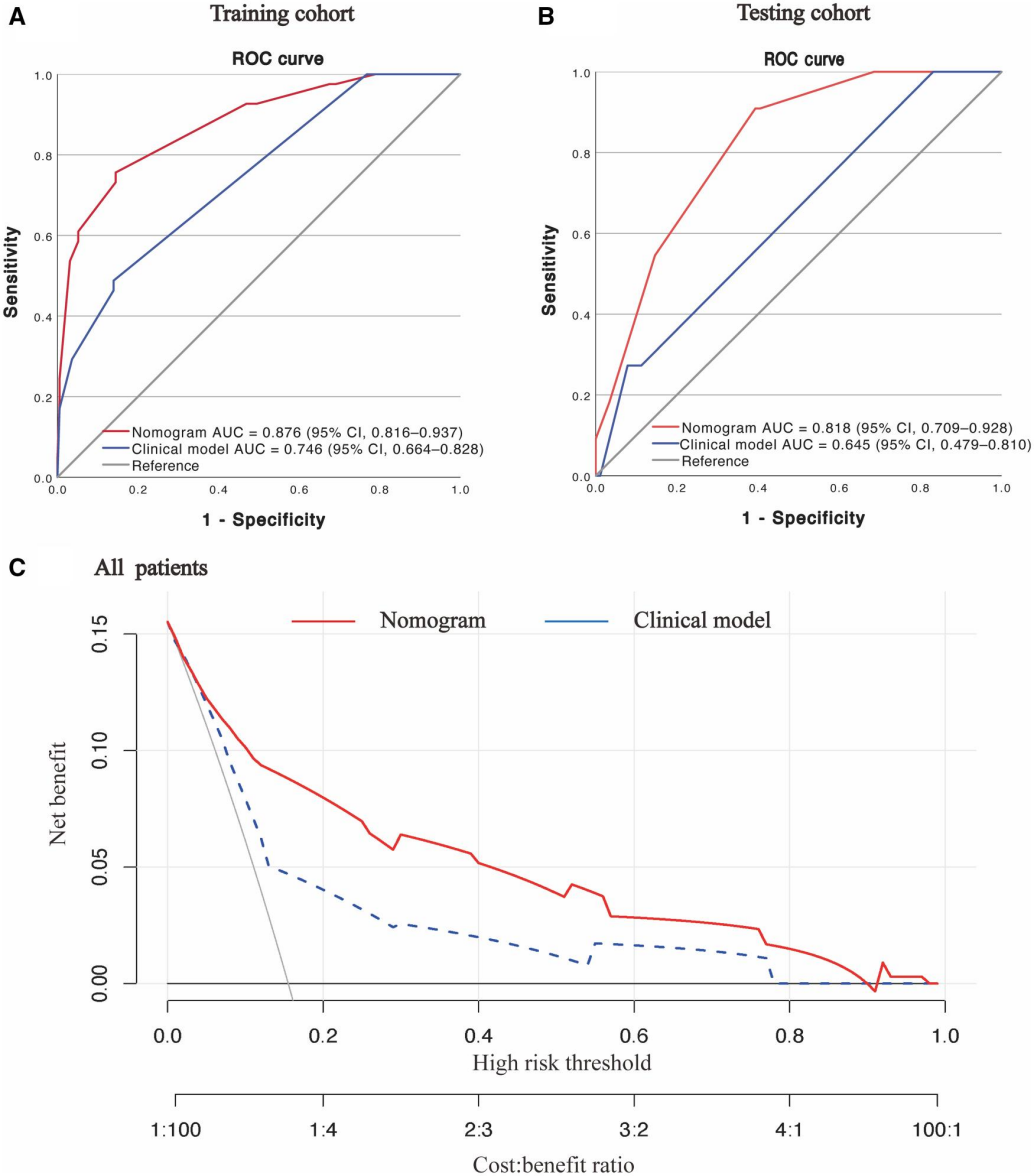
Comparison of prediction models for AS. (A) Receiver-operating characteristic (ROC) curves of the nomogram and the clinical model for predicting AS in the training cohort. (B) ROC curves of the nomogram and the clinical model for predicting AS in the testing cohort. (C) Decision curve analysis for the nomogram and clinical model in all 335 patients.

To demonstrate the superiority of the nomogram using the RICF score over the clinical model based on clinical characteristics, the RICF score was excluded and the clinical model was constructed based on the interval time from radiotherapy to surgery (OR, 2.808; 95% CI, 1.109–7.110, *P *=* *0.029), neoadjuvant radiotherapy (OR, 11.533; 95% CI, 1.149–91.812, *P *=* *0.021), and surgical approach (OR, 0.161; 95% CI, 0.075–0.346, *P *<* *0.001). The clinical model exhibited no multicollinearity (Supplementary Table S4). Regarding AS prediction, the clinical model achieved an AUROC of 0.746 (95% CI, 0.664–0.828) and 0.645 (95% CI, 0.479–0.810) in the training and testing cohorts, respectively. However, compared with the clinical model, the nomogram based on the RICF total score demonstrated a more robust ability to estimate the risk of AS in the training (DeLong test, *P *<* *0.001) and testing (DeLong test, *P *=* *0.018) cohorts, as shown in Figure 3A and B.

The clinical efficacy of the nomogram model based on the RICF score for predicting AS in all 335 patients was assessed by using DCA, as depicted in Figure 3C. The results demonstrated that using the aforementioned nomogram model to predict AS in patients with rectal cancer who underwent nCRT and sphincter-preserving surgery yielded greater net benefit than treat-all or treat-none strategies when the threshold probability ranged from 0 to 0.9.

## Discussion

In this study, the RICF score was developed to evaluate the severity of fibrosis in both the proximal and distal margins. Our findings revealed that the RICF total score was an independent risk factor for AS. Furthermore, we developed a nomogram for predicting the risk of AS in patients with rectal cancer undergoing nCRT and sphincter-preserving surgery, which incorporated the RICF total score, neoadjuvant radiotherapy, and surgical approach.

To date, no biomarker specific to RICF has been identified [21]. In patients with rectal cancer receiving nCRT, the primary consequence of RICF is the development of AS, which requires mandatory treatment [9, 22]. However, the severity of radiation-induced colorectal injury is currently assessed by using endoscopic techniques [23], computed tomography [24], and histopathological features [25, 26]. Studies have shown that the radiation-damage severity in patients with rectal cancer who underwent radiotherapy was inversely proportional to the length of the proximal margin from the tumor. Little damage was observed at the proximal margin >20 cm from the tumor [15, 16]. However, the length of anastomotic margins and AS in patients with rectal cancer who have received nCRT remains controversial. Therefore, considering fibrosis severity in anastomotic margins is crucial when identifying risk factors for benign AS after rectal cancer surgery [13, 27]. In this study, the RICF score was developed to distinguish the severity of fibrosis at the proximal and distal margins of patients with rectal cancer undergoing nCRT and sphincter-saving surgery.

Neoadjuvant radiotherapy is the cornerstone of comprehensive treatment for rectal cancer and has been identified as a risk factor for AS [10, 28]. During radiotherapy, the lower part of the rectum used for neorectum reconstruction is fixed and inevitably damaged by preoperative radiation [29]. Our data demonstrated that fibrosis was more severe at the distal margins than at the proximal margin, possibly due to the closer proximity of the distal margin to the tumor. Therefore, to preserve the function and control of the anal sphincter, it is essential to maintain the length of the distal margin as much as possible during sphincter-preserving surgery for primary tumor resection while still ensuring oncological safety. Galland *et al.* [30] found that the use of non-irradiated bowel for at least one end of an anastomosis significantly reduced the risk of anastomotic leakage. However, the optimal resection length of the proximal rectum remains controversial due to intestinal fibrosis and compliance after radiotherapy. Furthermore, we found a close correlation between the severity of fibrosis at the rectal stump and AS, and a marked increased risk of AS was associated with a higher RICF score. Extended resection of the proximal bowel with severe RICF may benefit patients with rectal cancer receiving preoperative radiotherapy. The RICF score may be applied to the proximal extended resection to prevent AS in patients with rectal cancer undergoing nCRT and sphincter-preserving surgery, which remains to be confirmed in further investigation.

Our findings revealed that neoadjuvant radiotherapy, surgical approach, and the RICF total score were identified as independent risk factors for AS in patients with rectal cancer who undergo nCRT and sphincter-preserving surgery. Previous studies have reported predisposing factors for AS in sphincter-preserving surgery, including anastomotic technique, anastomotic leakage, anastomotic distance, post-operative radiotherapy, pelvic sepsis, left colic artery preservation, and ileostomy [31–33]. Hu *et al.* [34] developed a nomogram based on left colic artery preservation, ileostomy, anastomotic leakage, and anastomotic distance to predict the risk of AS. Additionally, Singh *et al.* [35] found that anastomotic leakage triggered a robust inflammatory response in the surrounding tissue, which is a crucial pathological factor for AS. Although Kumar *et al.* [36] identified anastomotic leakage as a risk factor for AS, they found no association between ileostomy and AS. In our study, we found an association between AS and anastomotic leakage; however, it was not deemed an independent risk factor for AS. This difference could be attributed to confounding factors. Moreover, Zhang *et al.* [33] identified ileostomy and radiotherapy as critical factors associated with AS following anterior resection for rectal cancer. However, the relationship between diverting stoma and AS remains contentious. Babayev *et al.* [37] found that the long duration of ileostomy was associated with increased AS rates. A multivariate Cox model analysis concluded that there was no association between anastomotic leakage or ileostomy and the development of AS [6].

Our study identified open surgery as an independent risk factor for AS, possibly due to the increased tissue manipulation and trauma associated with open surgery. For example, Mathew *et al.* observed that, within 110 cases of benign AS, the prevalence of open surgery was notably high, reaching 71.8% [38]. First, laparotomy is a highly invasive procedure, resulting in a substantial systemic inflammatory response. This increased manipulation and trauma might have contributed to more significant tissue destruction, inflammation, and delayed healing at the anastomotic site, thereby elevating the likelihood of scar tissue formation and subsequent development of AS. Second, we use the ultrasonic scalpel more frequently in laparoscopic surgery than in the open procedure. Based on the preference of our surgical medical team, the electric knife in our centre is preferred over the ultrasonic blade in open surgery. The degree of thermal impact differs between the two approaches. However, whether this factor directly contributes to AS necessitates further investigation. Bressan *et al.* [39] reported that age of >71 years is a risk factor for AS. Our data indicated no association between AS and being >65 years of age. This study found that AS was associated with the time interval between radiotherapy and surgery rather than the chemotherapy regimen. Qin *et al.* [10] found that the chemotherapy regimen was not associated with the risk of AS. The impact of different chemotherapy regimens on rectal fibrosis has rarely been reported. However, prolonging the time interval between radiotherapy and surgery may increase the risk of rectal fibrosis [40].

Radiation exposure damages cells and tissues, leading to inflammatory and fibrotic reactions, with higher doses inducing more severe fibrotic lesions [41]. Radiotherapy is critical for local control in cases of locally advanced rectal cancer [42]. The FOWARC study demonstrated the importance of radiotherapy in treating rectal cancer, with reduced radiotherapy leading to a significant decrease in pathological complete response [43]. However, short-course radiotherapy with long intervals has been shown to have similar outcomes to conventional long-course radiotherapy [29]. Our findings suggest that short-course radiotherapy with a lower risk of AS can be a viable option for total neoadjuvant therapy, which is becoming more common with advances in neoadjuvant chemotherapy and immunotherapy [44]. We developed a nomogram for individualized assessment of the AS risk in patients with rectal cancer undergoing nCRT and sphincter-preserving surgery. Therefore, patients with rectal cancer should avoid long-course radiotherapy and open surgery to minimize the risk of excessive radiation exposure. Multiphoton imaging for fibrotic features compared with RICF scores may also be used for real-time assessment of anastomotic margins during rectal surgery [45].

This study has some limitations. First, our efforts are constrained by the retrospective nature of the analysis. Nevertheless, such retrospective studies frequently serve as precursors to more extensive prospective research. Consequently, further multicenter trial is necessary to confirm the external validity of our prediction model. Second, despite the occurrence of the possibility of delayed leakage and AS beyond a 2-year follow-up interval, this predictive model has shown good performance in predicting the risk of AS within 2 years after sphincter-preserving surgery; however, longer follow-up will be revealed in the future. Third, various validation methods are available for predictive models. However, the model using random grouping has also shown excellent performance for predicting AS in this study. Thus, we hope that cross-validation will be applied in our upcoming research. Fourth, the sample size might not be adequate for the TRIPOD statement; however, it was sufficient for calculating the expected sample size outlined by Riley *et al.* [46] (Supplementary Appendix S2). Thus, we hope that this limitation will be addressed in our future clinical trial. Fifth, as a retrospective study, this study did not provide data on whether splenic flexure mobilization and extended left hemicolectomy reduce the occurrence of anastomotic leakage and stenosis. However, we provided scientific data on whether the conventional 10-cm proximal fibrosis level RICF score affects the incidence of AS. This is crucial for patients, regardless of splenic flexure mobilization. Despite these limitations, to the best of our knowledge, this is the first predictive model based on the RICF score of anastomotic margins for the risk of AS in patients with rectal cancer undergoing nCRT and sphincter-preserving surgery.

## Conclusions

The RICF total score of the proximal and distal margins is an independent risk factor for AS in patients with rectal cancer undergoing nCRT and sphincter-preserving surgery. The prediction model we developed based on the RICF score is helpful for decision-making in tailored AS post-operative monitoring and early diagnosis.

## Supplementary Material

goae012_Supplementary_Data
