# Supplementary material for: Fibrosis signature of anastomotic margins for predicting anastomotic stenosis in rectal cancer with neoadjuvant chemoradiotherapy and sphincter-preserving surgery
Source: Gastroenterol Rep (Oxf). 2024 Mar 19;12:goae012. doi: 10.1093/gastro/goae012 (PMC10950477; doi:10.1093/gastro/goae012)
Supplement: goae012_Supplementary_Data [file goae012_supplementary_data.zip › 2023-171-Supplementary materials.docx]

**Supplementary Content**

**I: Figures**

**Figure S1.** Recruitment pathways for patients in the training and testing cohorts
**Figure S2.** The performance of the nomogram based on the RICF score for predicting AS

**Figure S3.** The performance of the clinical model for predicting AS

**II: Tables**

**Supplementary Table S1.** Consistency between double assessments for the RICF score

**Supplementary Table S2.** Clinical characteristics of patients in the training and testing cohort of this study

**Supplementary Table S3.** Multicollinearity assessment in the nomogram based on the RICF score

**Supplementary Table S4.** Multicollinearity assessment in the clinical model

**III: Appendix**

Appendix S1. Surgical procedures

Appendix S2. Sample size calculation for logistic regression analysis

This supplementary material has been provided by the authors to give readers additional information about their work.

**Figure S1**


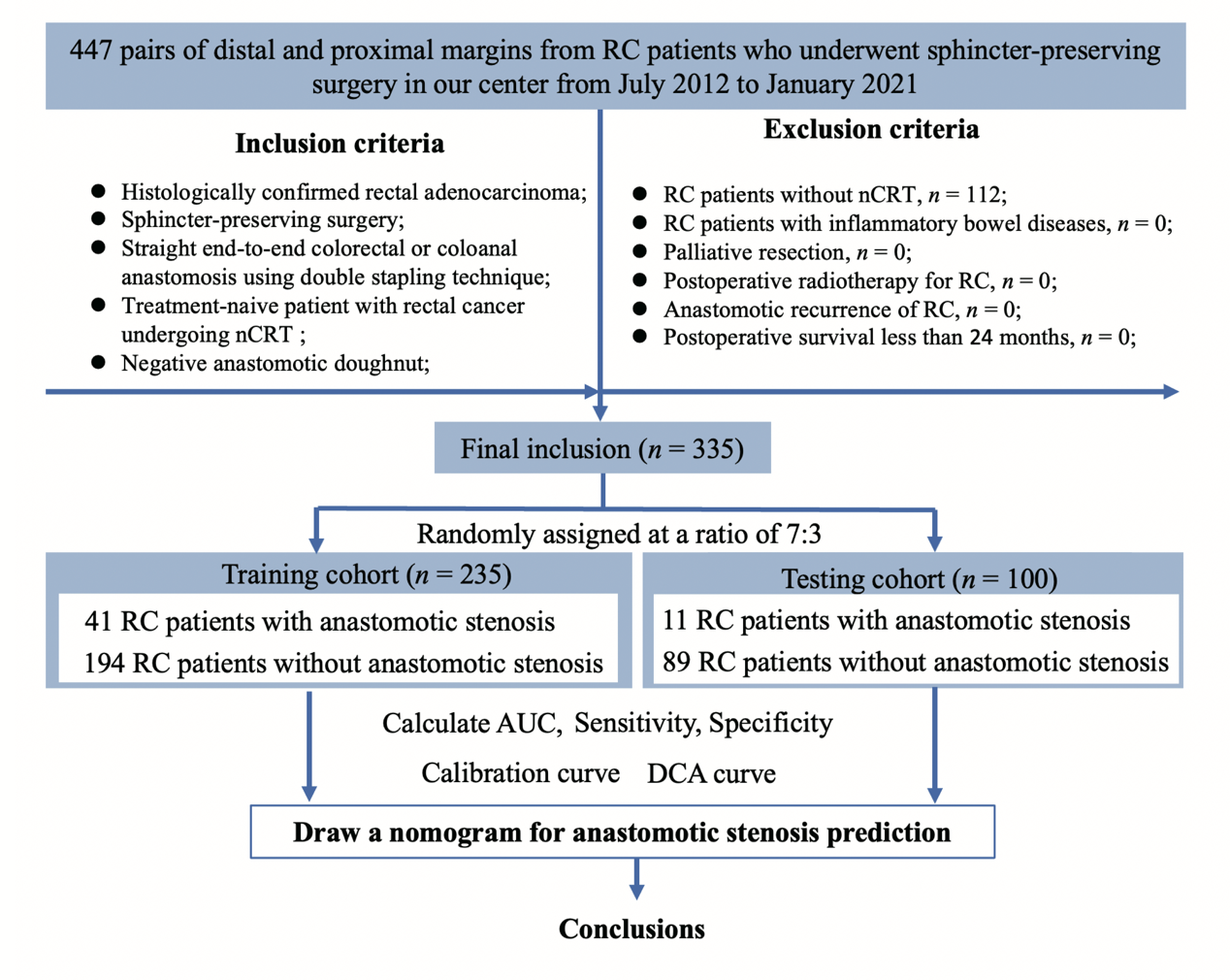


**Supplementary Figure S1. Recruitment pathways for patients in the training and testing cohorts**. A total of 335 proximal and distal margins from rectal cancer patients who were treated with neoadjuvant chemoradiotherapy (nCRT) and sphincter-preserving surgery at Fujian Medical University Union Hospital (FMUUH) from January 2012 to January 2021 were enrolled in this study.

Figure S2


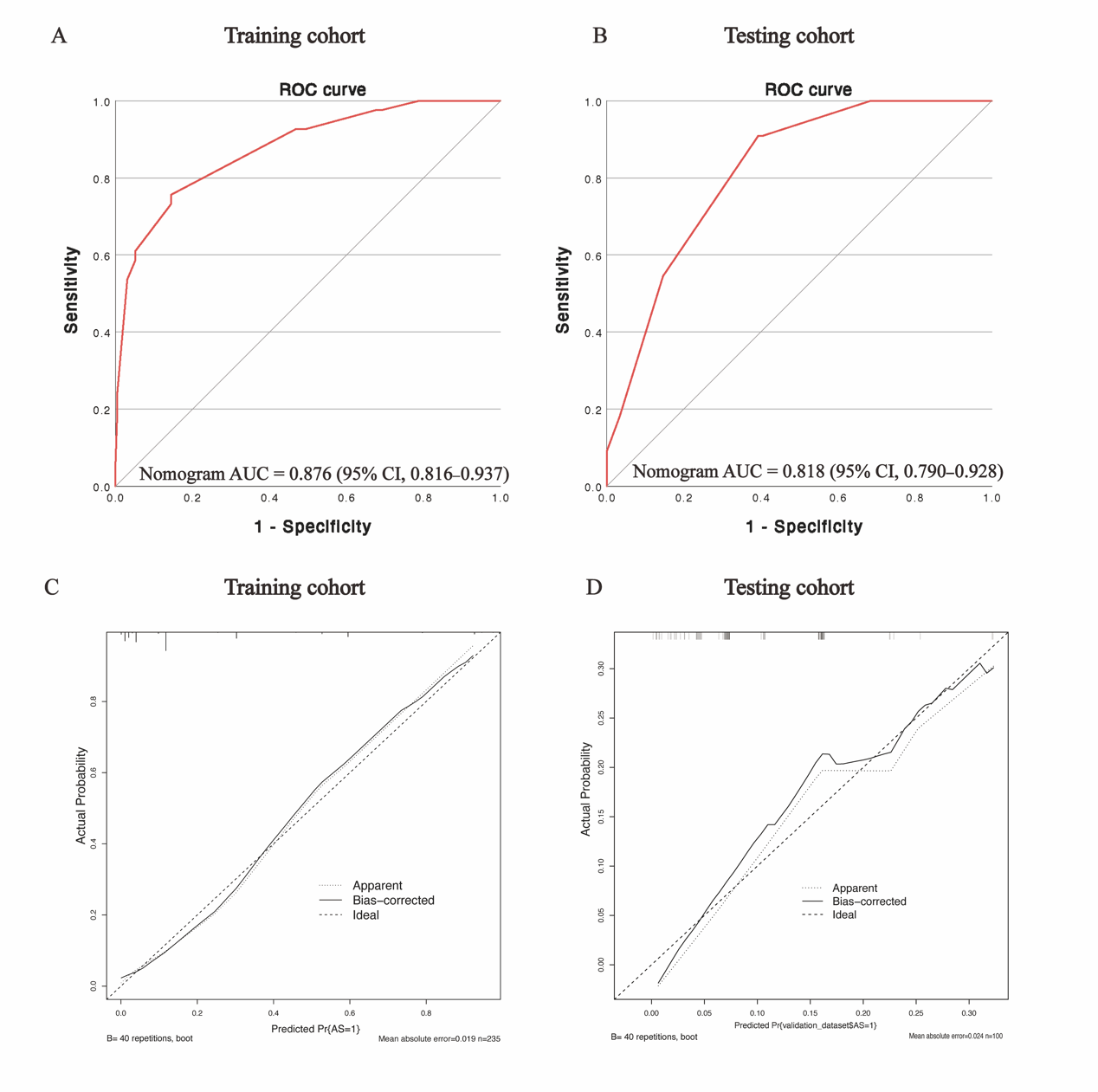


**Supplementary Figure S2. The performance of the nomogram based on the RICF score for predicting AS.** (A) The ROC curve of the nomogram for AS in the training cohort. (B) The ROC curve of the nomogram for AS in the testing cohort. (C) Calibration curve of the nomogram in the training cohort. (D) Calibration curve of the nomogram in the testing cohort.

| **Supplementary Table S1. Consistency between double assessments for the RICF score** | | | | |
| --- | --- | --- | --- | --- |
| Variable | Dr. M.X. | Dr. Q.Y. | ICC | 95% CI |
| Proximal margin | 1.6 ± 0.8 | 1.6 ± 0.8 | 0.809 | 0.758-0.916 |
| Distal margin | 2.1 ± 0.6 | 2.0 ± 0.6 | 0.846 | 0.795-0.898 |
| The RICF total score | 3.7 ± 1.5 | 3.7 ± 1.5 | 0.812 | 0.772-0.852 |
| Data are expressed as mean ± standard deviation. RICF = radiation-induced colorectal fibrosis. | | | | |

| **Supplementary Table S2. Clinical characteristics of patients in the training and testing cohort of this study** | | | |
| --- | --- | --- | --- |
| **Characteristic** | **Training cohort**  (*n* = 235) | **Testing cohort**  (*n* = 100) | *P* |
| Age (<65 years/≥65 years) | 150 (66.8)/80 (33.2) | 66 (66.0)/34 (34.0) | 0.805 |
| Sex (Male/Female) | 161 (68.5)/74 (31.5) | 72 (72.0)/28 (28.0) | 0.604 |
| BMI (<25/≥25) | 157 (66.8)/78 (33.2) | 68 (68.0)/32 (32.0) | 0.899 |
| Drinking | 86 (36.6)/149 (63.4) | 46 (46.0)/54 (54.0) | 0.114 |
| Smoking | 88 (37.5)/147 (62.5) | 48 (48.0)/52 (52.0) | 0.089 |
| Diabetes | 18 (7.7)/217 (92.3) | 11 (11.0)/89 (89.0) | 0.395 |
| Hypertension | 46 (19.56)/189 (80.4) | 23 (23.0)/77 (77.0) | 0.465 |
| pT Stage (T0-2/T3-4) | 109 (46.4)/126 (53.6) | 54 (54.0)/46 (46.0) | 0.863 |
| pN Stage (N0/N+) | 176 (74.9)/59(25.1) | 83 (83.0)/17 (17.0) | 0.118 |
| pM Stage (M0/M1) | 219 (93.2)/16 (6.8) | 89 (89.0)/11 (11.0) | 0.196 |
| AJCC Stage (0-II /III-IV) | 172(73.2)/63(26.8) | 73 (73.0)/27 (27.0) | 0.971 |
| TRG (0/1-3) | 46(19.6)/189(80.4) | 24 (24.0)/78 (78.0) | 0.456 |
| Neoadjuvant chemotherapy (XELOX/others) | 173 (73.6)/62 (26.4) | 70 (70.0)/30 (30.0) | 0.506 |
| Neoadjuvant radiotherapy (25 Gy, 5 F/50.4 Gy, 25 F) | 46 (20.0)/189 (80.0) | 18 (18.0)/82 (82.0) | 0.764 |
| Interval time from radiotherapy to surgery  (<10 weeks/≥10 weeks) | 40 (17.1)/195 (82.9) | 9 (9.0)/91 (91.0) | 0.065 |
| Tumor Location (Low/Mid-Upper) | 140 (59.6)/95 (40.4) | 63 (63.0)/37 (37.0) | 0.625 |
| Intraoperative bleeding (mean ± SD, mL) | 67 ± 121 | 57 ± 33 | 0.437 |
| Length of surgery (mean ± SD, min) | 263 ± 71 | 259 ± 65 | 0.633 |
| Total lymph nodes harvested (LNs < 12/LNs ≥ 12) | 89 (37.9)/146 (62.1) | 45 (45.0)/55 (55.0) | 0.226 |
| Surgical approach (Open/MIS) | 20 (8.5)/215 (91.5) | 4 (4.0)/96 (96.0) | 0.170 |
| Ileostomy | 125 (53.2)/110 (46.8) | 61 (61.0)/39 (39.0) | 0.230 |
| Anastomotic leakage | 38 (16.2)/197 (83.8) | 11 (11.0)/89 (89.0) | 0.242 |
| Anastomotic stenosis | 41 (17.5)/194 (82.5) | 11 (11.0)/89 (89.0) | 0.186 |
| BMI = body mass index, AJCC = The American Joint Committee on Cancer, TRG = tumor regression grade, XELOX = capecitabine plus oxaliplatin, F= fractions, SD = standard deviation, LN = lymph node, MIS = minimally invasive surgery. | | | |

| **Supplementary Table S3. Multicollinearity assessment in the nomogram based on the RICF score** | | |
| --- | --- | --- |
| Predictor | Collinearity statistics | |
|  | Tolerance | Variance inflation factor |
| Neoadjuvant radiotherapy (50.4 Gy, 25 F/25 Gy, 5 F) | 0.977 | 1.023 |
| Surgical approach (Open vs MIS) | 0.941 | 1.063 |
| RICF total score | 0.938 | 1.066 |
| RICF = radiation-induced colorectal fibrosis; MIS = minimally invasive surgery. | | |

| **Supplementary Table S4. Multicollinearity assessment in the clinical model** | | |
| --- | --- | --- |
| Predictor | Collinearity statistics | |
|  | Tolerance | Variance inflation factor |
| Interval time from radiotherapy to surgery  (<10 weeks/≥10 weeks) | 0.989 | 1.011 |
| Neoadjuvant radiotherapy (50.4 Gy, 25 F/25 Gy, 5 F) | 0.993 | 1.019 |
| Surgical approach (Open vs MIS) | 0.995 | 1.005 |
| F= fractions, MIS = minimally invasive surgery. | | |

**Appendix S1. Surgical procedures**

In this study, radical sphincter-preserving surgery was conducted on rectal cancer patients, adhering to the principles of total mesorectal excision (TME) [1, 2]. The surgical approaches included both laparoscopic and open methods. In all patients, high ligation of the inferior mesenteric artery was performed. Proximal division of the mesentery along the left Toldt’s space allowed the descending colon to reach the anus without any tension. The dissociation of the splenic flexure is determined according to the length of the sigmoid colon. For the anastomosis procedure, an end-to-end technique was employed using a stapling device. Standard mechanical bowel preparation was conducted, and an abdominal drainage tube was inserted. The consideration and strategic implementation of an ileostomy were based on factors including the level of anastomosis, its integrity, the presence of comorbidities, and the patient's nutritional status.

**Appendix S2. Sample size calculation for logistic regression**

There are various methods used to calculate sample size. One was the transparent reporting of a multivariable prediction model for individual prognosis or diagnosis (TRIPOD) statement, a well-known rule of thumb for the required sample size is to ensure at least 10 events for each predictor parameter [3]. However, some researchers suspected that 10 EPV was too lenient or too strict [4, 5]. The sample size would be predetermined on statistical grounds only for a planned prospective prediction model development study. The other was calculating the expected sample size based on the paper by Riley et al [6]. For a binary outcome van Smeden et al. [7] use simulation, across a range of scenarios, to derive an approximation of the expected average error in the outcome probabilities when a derived model is applied to new individuals from the target population. Their derived formula was originally developed based on 12 or fewer predictor parameters, but they have since updated the simulations to allow for 30 or fewer predictor parameters. The derived formula is:

In (MAPE) = -0.508-0.544In(n)+ 0.259In(Ø)+ 0.504In(P)

Here, n is the sample size of the development dataset, Ø is the anticipated outcome proportion (<0.5), and P is the number of candidate predictor parameters (<30). MAPE denotes the Mean Absolute Prediction Error (i.e., the average error in the model's estimated outcome probability one would allow for in the intended setting of application of the model). Rearranging this equation, and choosing a target value for MAPE, MAPE is no larger than 0.050, but lower values might be appropriate in settings when precise predictions are demanded if the consequences of wrong decisions are large. the required sample size is:

$$n=exp\left( \frac{-0.508-0.544In(n)+ 0.259In(Ø)+ 0.504In(p)}{0.544} \right)$$

In this study, we set MAPE to 0.050, AS with an anticipated outcome proportion of 0.078 [8] and 7 candidate predictor parameters (logistic regression multivariate analysis, variable < 0.05). Thus at least 175 participants (about 14 events) in the development dataset. Our sample size might be not adequate for the TRIPOD method but was enough for the expected sample size based on the paper by Riley et al. Thus, we hope that this limitation will be solved in our upcoming clinical trial. Fewer studies have been reported for sample size calculation in the validation cohort. Lei et al.[9] revealed that the ratio between the primary and testing cohorts was 7:3. In our study, the testing cohort contained 100 patients, which was enough to validate the prediction model.

**References**

1. Jiang WZ, Xu JM, Xing JD, Qiu HZ, Wang ZQ, Kang L, et al. Short-term Outcomes of Laparoscopy-Assisted vs Open Surgery for Patients With Low Rectal Cancer: The LASRE Randomized Clinical Trial. JAMA Oncol. 2022 Sep 15.

2. Chinelli J, Rodriguez G. Laparoscopic Low Anterior Resection and Total Mesorectal Excision. Dis Colon Rectum. 2023 May 1;66(5):e220-e21.

3. Collins GS, Reitsma JB, Altman DG, Moons KG. Transparent Reporting of a multivariable prediction model for Individual Prognosis or Diagnosis (TRIPOD): the TRIPOD statement. Ann Intern Med. 2015 Jan 6;162(1):55-63.

4. Vittinghoff E, McCulloch CE. Relaxing the rule of ten events per variable in logistic and Cox regression. Am J Epidemiol. 2007 Mar 15;165(6):710-8.

5. Wynants L, Bouwmeester W, Moons KG, Moerbeek M, Timmerman D, Van Huffel S, et al. A simulation study of sample size demonstrated the importance of the number of events per variable to develop prediction models in clustered data. J Clin Epidemiol. 2015 Dec;68(12):1406-14.

6. Riley RD, Ensor J, Snell KIE, Harrell FE, Jr., Martin GP, Reitsma JB, et al. Calculating the sample size required for developing a clinical prediction model. BMJ. 2020 Mar 18;368:m441.

7. van Smeden M, Moons KG, de Groot JA, Collins GS, Altman DG, Eijkemans MJ, et al. Sample size for binary logistic prediction models: Beyond events per variable criteria. Stat Methods Med Res. 2019 Aug;28(8):2455-74.

8. Lee SY, Kim CH, Kim YJ, Kim HR. Anastomotic stricture after ultralow anterior resection or intersphincteric resection for very low-lying rectal cancer. Surg Endosc. 2018 Feb;32(2):660-66.

9. Lei Z, Li J, Wu D, Xia Y, Wang Q, Si A, et al. Nomogram for Preoperative Estimation of Microvascular Invasion Risk in Hepatitis B Virus-Related Hepatocellular Carcinoma Within the Milan Criteria. JAMA Surg. 2016 Apr;151(4):356-63.
